# Development of Wearable Devices for Collecting Digital Rehabilitation/Fitness Data from Lower Limbs

**DOI:** 10.3390/s24061935

**Published:** 2024-03-18

**Authors:** Yu-Jung Huang, Chao-Shu Chang, Yu-Chi Wu, Chin-Chuan Han, Yuan-Yang Cheng, Hsian-Min Chen

**Affiliations:** 1 Department of Electrical Engineering, National United University, Miaoli 36003, Taiwan; yujungzip@gmail.com; 2 Department of Information Management, National United University, Miaoli 36003, Taiwan; cschang@nuu.edu.tw; 3 Department of Computer Science and Information Engineering, National United University, Miaoli 36003, Taiwan; cchan@nuu.edu.tw; 4 Department of Physical Medicine and Rehabilitation, Taichung Veterans General Hospital, Taichung City 40705, Taiwan; rifampin@gmail.com; 5 Center for Quantitative Imaging in Medicine (CQUIM), Department of Medical Research, Taichung Veterans General Hospital, Taichung City 40705, Taiwan; hsmin6511@gmail.com

**Keywords:** rehabilitation, fitness activity recognition, wearable devices, radio frequency, inertial measurement unit, machine learning

## Abstract

Lower extremity exercises are considered a standard and necessary treatment for rehabilitation and a well-rounded fitness routine, which builds strength, flexibility, and balance. The efficacy of rehabilitation programs hinges on meticulous monitoring of both adherence to home exercise routines and the quality of performance. However, in a home environment, patients often tend to inaccurately report the number of exercises performed and overlook the correctness of their rehabilitation motions, lacking quantifiable and systematic standards, thus impeding the recovery process. To address these challenges, there is a crucial need for a lightweight, unbiased, cost-effective, and objective wearable motion capture (Mocap) system designed for monitoring and evaluating home-based rehabilitation/fitness programs. This paper focuses on the development of such a system to gather exercise data into usable metrics. Five radio frequency (RF) inertial measurement unit (IMU) devices (RF-IMUs) were developed and strategically placed on calves, thighs, and abdomens. A two-layer long short-term memory (LSTM) model was used for fitness activity recognition (FAR) with an average accuracy of 97.4%. An intelligent smartphone algorithm was developed to track motion, recognize activity, and calculate key exercise variables in real time for squat, high knees, and lunge exercises. Additionally, a 3D avatar on the smartphone App allows users to observe and track their progress in real time or by replaying their exercise motions. A dynamic time warping (DTW) algorithm was also integrated into the system for scoring the similarity in two motions. The system’s adaptability shows promise for applications in medical rehabilitation and sports.

## 1. Introduction

According to the research report of the World Health Organization (WHO), the proportion of elderly people with disabilities aged over 65 is close to 40%. In addition, the data of the WHO also point out that about 1 billion people around the globe suffer from some degree of life impairment due to neurodegenerative diseases, cerebrovascular diseases, neurological injuries, etc. Therefore, the WHO suggests that the development of relevant rehabilitation assistive technology, support services, and social security and inclusive policies to help the elderly, the disabled, and other people in need to return to a healthy, comfortable, and dignified lifestyle should be the focus of attention of advanced countries around the world [1].

Maintaining proper posture and appropriate intensity of rehabilitation training is extremely important for patients after surgery due to aging, trauma, disease, stroke, or sports injuries. Within six months after the onset of cerebral stroke, through accurate and appropriate rehabilitation training, about 70–80% of patients can regain the ability to walk, and about 50–66% can live independently and have a certain quality of life [2]. However, the time and labor required for rehabilitation training are considerable, and the general shortage of medical personnel in Taiwan and some other countries in recent years has made it difficult to provide one-on-one rehabilitation or timely orthopedic treatment. In addition, the assessment of patients’ recovery after rehabilitation is mostly based on visual inspection by doctors, and the collection of objective rehabilitation posture data has not yet become widespread, exacerbating the dilemma of unmet rehabilitation needs. In addition to the lack of experienced doctors, other factors, such as medical funding, rehabilitation resources, and relatively expensive costs, have also led to severe difficulties faced by those in need of rehabilitation. Therefore, the WHO launched the Rehabilitation 2030 Initiative to address this issue [3].

The conventional rehabilitation approach involves initial guidance by physical therapists in a hospital, followed by patients regularly performing rehabilitation exercises at home, and ensuring the effectiveness of rehabilitation programs involves monitoring both the adherence to home exercise routines and the quality of performance. Home exercise performance is typically assessed post hoc through patient self-reports, journals, and logs or through evaluator scores of participant performance during videotaped or live sessions, which are likely to be subject to significant bias and inaccuracy [4]. In the home environment, patients tend to over-report the number of exercises performed per session or often overlook the correctness of their rehabilitation motions, lacking quantitative and systematic standards and thus slowing down the recovery process. To mitigate these challenges, a lightweight, unbiased, low-cost, and objective wearable motion capture (Mocap) rehabilitation system is needed for monitoring and evaluating home-based rehabilitation programs.

In rehabilitation exercises, lower extremity exercises are considered a standard and necessary treatment for post-surgical rehabilitation [5,6,7], such as total hip replacement or total knee replacement, or for the treatment of musculoskeletal disorders of the lower extremities, such as osteoarthritis (OA), and they are also an important part of a well-rounded fitness routine that builds strength, flexibility, and balance. Hence, there are wearable devices developed for data acquisition for lower limb rehabilitation and fitness posture.

Thanks to the proliferation of the Internet of Things (IoT) [8,9,10] and novel low-cost micro-electro-mechanical system (MEMS) inertial measurement units (IMUs), there has been a rise in mobile wearable devices. These devices play a pivotal role in capturing physiological data such as heart rate and exercise status. Consequently, the medical and healthcare sector stands out as a prominent domain for their applications [11]. The convergence of IoT technology with medical sensing devices and wearable technologies allows for seamless integration. This integration not only enhances the quality and efficiency of services within the healthcare field [12] but also supports healthcare organizations in the effective management of medical information and resources [13,14].

In recent years, there has been an increasing number of studies on IMU-based Mocap in the fields of rehabilitation and fitness that take advantage of IMU’s portability, easy deployment, and affordability. Komaris et al. [4] developed a wearable system to assess therapeutic movement using a single IMU to derive characteristics of movement smoothness based on log dimensionless jerk, movement intensity based on the Euclidean norm of the acceleration, movement regularity based on autocorrelation, and movement stability based on dynamic time warping (DTW). Several healthy participants were tested for week-long strengthening and cardiorespiratory endurance exercises (knee extension supine, split squad, calm advanced, half squad, and mountain climber), targeting the main muscle groups of the lower limbs and core. However, no AI algorithms were proposed for motion recognition in this system; therefore, it does not have the capability to automatically identify the type of exercises. Chen et al. [15] proposed an IMU-based rehabilitation system for both the upper and lower limbs. Two commercially available wearable IMU sensors from Gyro Systems, Inc. (Zhubei City, Taiwan) were used to detect rehabilitation motions (climbing, pendular, and pulling towel exercises for upper limbs; knee-to-chest and hip-abduction exercises for lower limbs) in patients with specific conditions, and the data were transmitted to an Android App for correctness assessment and statistical analysis. Different conditions were used for judging different rehabilitation motions, for example, the knee-to-chest exercise was recognized when the thigh and knee angles were greater than a certain threshold; therefore, the classification was coarse and needed fine-tuning for these thresholds. Their experimental results demonstrate average errors of less than 5° for knee and elbow angles, with recognition rates exceeding 85% for all rehabilitation exercises. Wu et al. [16] introduced a multi-procedure intelligent algorithm for weight training utilizing two IMUs. The first procedure tracked motion, estimated arm orientation, and calculated wrist and elbow positions. The second procedure employed deep learning for posture recognition. The final procedure determined exercise prescription variables, inferring the user’s exercise state, triggering corresponding events, and calculating key indicators of weight training exercises in real time. Their experimental verification tests demonstrate accurate estimation and posture recognition, with an accuracy of 99% for the presented system. However, this system does not provide a measure for the similarity or stability in motions from different persons or different motion cycles, e.g., coach’s motion vs. trainee’s motion. Lin et al. [17] presented an IMU-aided fitness system utilizing at most three wearable nine-axis IMU sensors developed by Gyro Systems, Inc. to detect three kinds of exercises including squats, bridges, and double leg raises. The system used roll angles of sensors at different positions to measure the correctness of exercises. The roll angles had to satisfy a sequence of criteria in order to be qualified as a correct exercise. Six participants were instructed to conduct three kinds of exercises for 7 days, resulting in three groups of 10-time squats, bridges, and double leg raises, respectively, every day. Their experimental findings indicate that users have the potential to enhance their skills with prolonged usage of the system. However, no AI algorithms are proposed for motion recognition in this system. Schlage et al. [18] introduced a cost-effective IMU-based system for capturing human motion sequences, realized by a Stickman model, and deriving three joint angles (flexion/extension, rotation, abduction/adduction) of the lower extremities to detect malposition. The IMU used in the system is commercially available from XSens Dots [19]. Three activities, including squatting, walking, and climbing stairs (up and down), were performed by only one participant. The measured angles were compared to two commercial systems, including Qualisys [20] and KneTex [21], using DTW. The results of flexion/extension (F/E) show a high accuracy, while rotation (Rot) and abduction/adduction (A/A) show a higher deviation. In this system, neither motion recognition nor an AI algorithm is proposed. Müller et al. [22] addressed the fitness activity recognition (FAR) task and designed a scaling fully convolutional network (scaling-FCN) in addition to three existing convolutional neural networks (CNNs) for FAR using IMU data. FAR can present unique challenges to the human activity recognition task (HAR), including greater similarity between individual activities and fewer available data for model training. An IMU data set of 20 participants for seven different running exercises was recorded including the following: regular running, side skips (right and left direction), Carioca running (right and left direction), heel-to-butt running, and high-knee running. A total of four IMUs were worn on the participant’s body, one on each ankle and each wrist. Their results indicate that CNNs are generally well-suited for FAR. Notably, significant performance improvements can be attained through selective sensor removal. However, it is noteworthy that traditional machine learning (ML) architectures can still rival or even surpass CNNs, particularly when leveraging favorable input data.

From the above discussion, it is found that IMU-based at-home rehabilitation or fitness systems need to be low-cost, automatically recognize the type of exercise, and gather exercise data into usable metrics. In this paper, which is partially based on our previous work [16], we integrated hardware and software systems to develop attitude and heading reference system (AHRS) devices for lower extremity motion capture and an intelligent algorithm that performs motion tracking and posture recognition and calculates key exercise indexes for three different exercises (squats, high knees, and lunges). Five devices with radio frequency (RF) and IMUs were developed and used in the system, one each on the right and left calves, one each on the right and left thighs, and one on the abdomen. A two-layer long short-term memory (LSTM) model was used for training exercise data; therefore, the FAR was achieved. Furthermore, a smartphone App was developed on which Unity’s Animation System was used to display 3D humanoid movements. Users could have a real-time display or a replay of their exercise motions, monitoring their exercise progress. A DTW algorithm was also integrated into the system for scoring the similarity in two motions. Therefore, the proposed system, different from the other systems in the literature, relies on the following aspects:

- Cost-effective RF modules were developed to implement the proposed system for transmitting the motion data from each IMU at a sampling rate of 60 Hz, while most other systems used Wi-Fi or Bluetooth. RF communication provides more reliable data acquisition than Bluetooth or Wi-Fi in crowded environments where several Wi-Fi and Bluetooth networks coexist.
- A custom do-it-yourself IMU-based system that does not use commercial IMU systems is presented.
- The developed system was tested in the laboratory environment in real time using a 3D avatar to represent 3D movement.
- A pre-trained machine learning model deployed on the smartphone can instantly obtain FAR (fitness activity recognition) results and display fitness activity data such as repetitions, intensity, energy consumption, and exercise duration, leveraging the data generated by users during fitness/rehabilitation to provide instantaneous and personalized insights.
- A DTW algorithm was integrated into the system for scoring the similarity in two motions.

## 2. Materials and Methods

**Figure 1:**
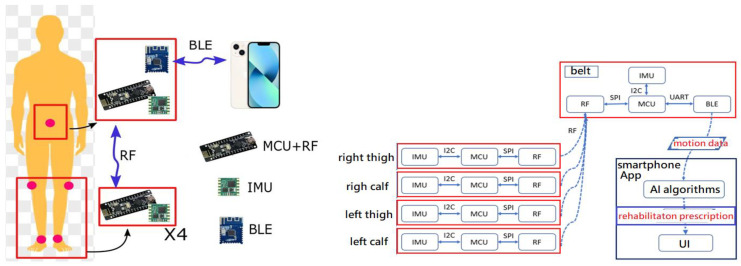
System architecture ((**left**): wearing positions of devices (red dots), (**right**): schematic system diagram).

The proposed system architecture is shown in Figure 1. In order to facilitate the sampling of lower limb exercise motion data, we developed four RF-IMU wearable devices and one RF-IMU-BLE (BLE, Bluetooth Low-Energy) device, using IMUs, RF+ MCUs (MCU, micro controller units), and BLE+MCU. We also developed a smartphone App to perform real-time recognition of lower limb rehabilitation exercises, to present rehabilitation actions with a virtual humanoid model (Unity), and to compute quantitative data on lower limb rehabilitation exercises. After data collection, the 9-axis raw data were converted into quaternions through a series of calculations based on AHRS, and then a virtual humanoid model (Unity) was used on the smartphone to recreate the motion and present the rehabilitative movements in real time. The machine learning algorithm, two-layer LSTM (long short-term memory), was used for FAR, while DTW was used for motion comparison (motion similarity scoring).

### 2.1. Hardware

The hardware devices are mainly composed of the following modules:

1. Microcontroller unit (MCU) with BLE: It controls the reading of IMU data, programs algorithms to convert values, and exchanges data through Bluetooth. ESP32 (Espressif Systems, Shanghai, China) is used in this system.
2. Inertial measurement unit (IMU): It captures the spatial coordinate vector data (quaternion) of motion attitude (9 axes including 3-axis acceleration, 3-axis angular velocity, and 3-axis geomagnetism). BNO055 (Bosch Sensortec GmbH, Reutlingen, Germany) is used in this system, which can also output stable quaternion data in addition to 9-axis data.
3. RF with MCU: RF-Nano (Arduino Nano R3 + nRF24L01) is used in this system to transmit data wirelessly using RF. It combines the simplicity and compatibility of Arduino Nano R3′s ATmega328 with the usefulness of the nRF24L01+ (Nordic Semiconductor ASA, Trondheim, Norway) 2.4 GHz radio transceiver in one single board [23]. nRF24L01+ is a compact 2.4 GHz transceiver chip featuring an integrated baseband protocol engine known as Enhanced ShockBurst™, ideal for energy-efficient wireless applications [24]. Engineered to function within the globally recognized ISM frequency band of 2.400–2.4835 GHz, nRF24L01+ offers versatility and reliability. The specifications of the MCU in RF-Nano are identical to the Arduino Nano R3 development board. The nRF24L01+ chip is connected to the ATmega328P chip directly on the board. This means there are SPI pins on the GPIO that you can no longer use for other purposes. These pins are listed in Table 1. The MCU is connected to the IMU via the I2C to read the motion data.
4. Lithium battery charging module: It provides power for each individual module.

**Figure 2:**
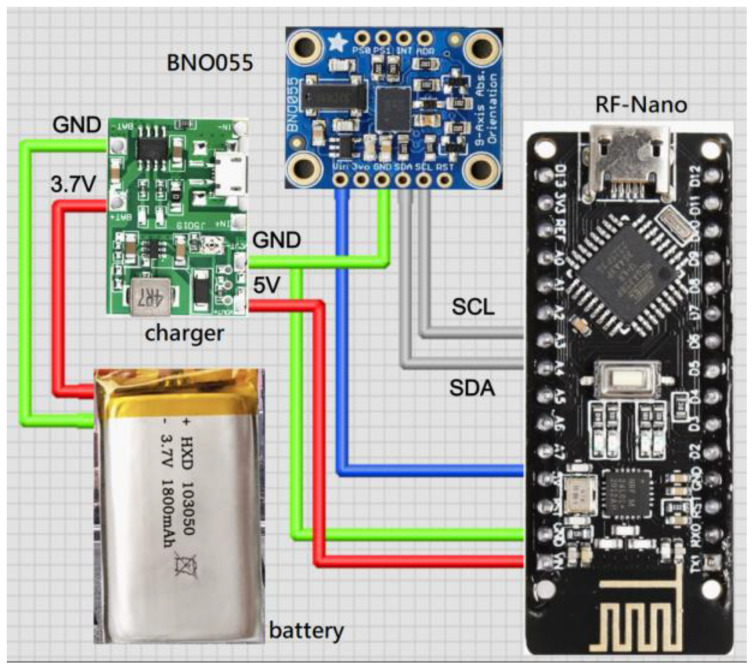
Connection diagram of RF-IMU.

**Figure 3:**
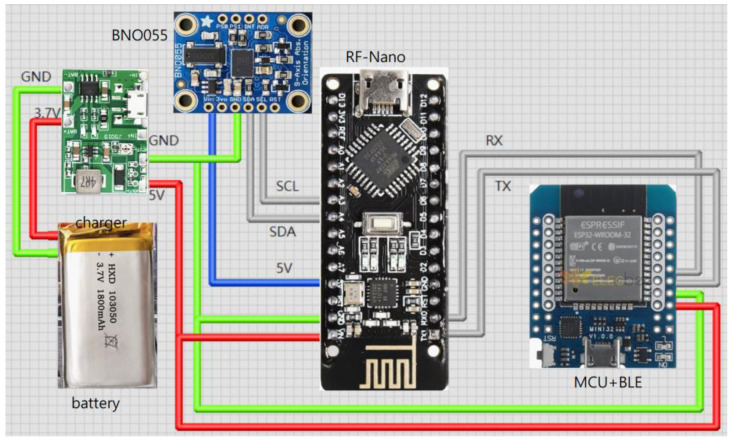
Connection diagram of RF-IMU-BLE.

**Figure 4:**
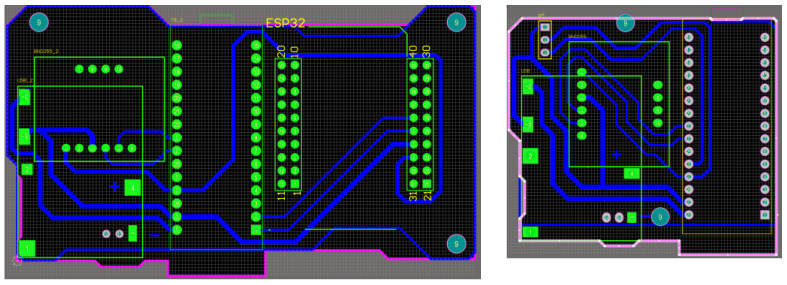
Circuit boards (**right**) RF-IMU-BLE and (**left**) RF-IMU.

The wearing positions of the developed devices for capturing the lower limb exercises are shown in the left diagram in Figure 1. Four sets of motion sensing devices (RF-IMUs) of the same design were worn on the calves and thighs of the two lower limbs, and one motion sensing device (RF-IMU-BLE) was worn on the abdomen. The purpose of using the RF-IMU-BLE on the abdomen is to provide the motion of the upper torso and a reference point for the other four RF-IMUs. When the exercise involves the upper torso moving forward, bending, or turning right/left, we need an IMU on the abdomen to detect those motions. The RF-IMU worn on the lower limb includes an RF transmission module, a microcontroller, and an IMU, where the microcontroller unit (MCU) communicates with the RF module via the SPI (Serial Peripheral Interface) and the MCU communicates with the IMU via the I2C (Inter-Integrated Circuit), as shown in Figure 2. The RF-IMU-BLE on the abdomen consists of an RF module, an MCU, an IMU, and a BLE module. The MCU communicates with the RF module and the IMU in the same way as in the device (RF-IMU) worn on the lower limb, while the MCU communicates with the BLE module through the UART (Universal Asynchronous Receiver/Transmitter), as shown in Figure 3. The Bluetooth module is responsible for transmitting the collected motion data from the lower limbs and the abdomen to the smartphone, and finally, the smartphone App performs FAR and motion similarity algorithms and displays the Unity humanoid in the user interface. The schematic system structure is shown in the right diagram in Figure 1. Figure 4 shows the circuit boards for the RF-IMU-BLE device (right) and the RF-IMU device (left).

**Figure 5:**
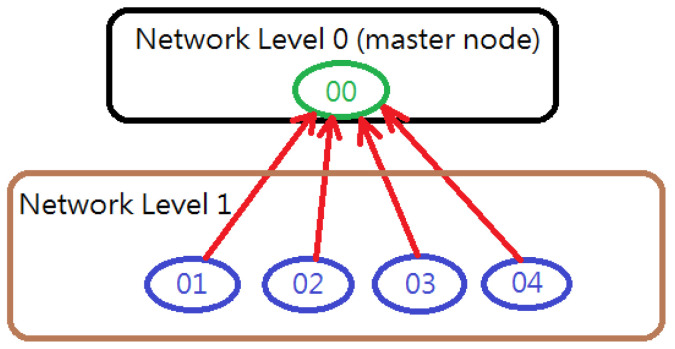
RF communication topology.

The proposed system adopts wireless RF communication so that there is no wire-hanging problem. Unidirectional data transmission is utilized to continuously update the motion data stored inside the MCU at the fastest speed. The nRF24L01+ chip can use multiple channels for communication. Each has 1 channel (ID 0) for transmitting data and 5 channels (ID 1–ID 5) for receiving data. Figure 5 illustrates the RF communication topology used in the presented system. The RF-IMU-BLE device (master node) is in charge of receiving data from the other four RF-IMU devices; therefore, it is set up to have four channels (ID 1–ID 4) for receiving data and no channel for transmitting data. The RF-IMU device is in charge of only transmitting data to the RF-IMU-BLE; therefore, it is set up to have one channel for sending data and no channel for receiving data. Normally, to establish a communication link at the start of the communication before full communication, the master device and slave device use a handshaking mechanism. Since the handshaking between each RF-IMU device and RF-IMU-BLE takes time and we have four RF-IMU devices, the handshaking time spent on 4 channels would slow down the data sampling rate. Therefore, handshaking is not used in the presented system. The data rate of nRF24L01+ is configured at 1 Mbps (Megabit per second) by the MCU.

The MCU in the RF-IMU reads the IMU data and then transmits the data via RF to the RF-IMU-BLE on the abdomen. The RF-IMU-BLE transmits the received data to the smartphone via Bluetooth. The RF module in the RF-IMU-BLE does not transmit data. It is only responsible for receiving data. The ESP32 (MCU with BLE) is responsible for controlling Bluetooth transmission, and it operates independently from the MCU responsible for transmitting/receiving RF data.

### 2.2. Software

**Figure 6:**
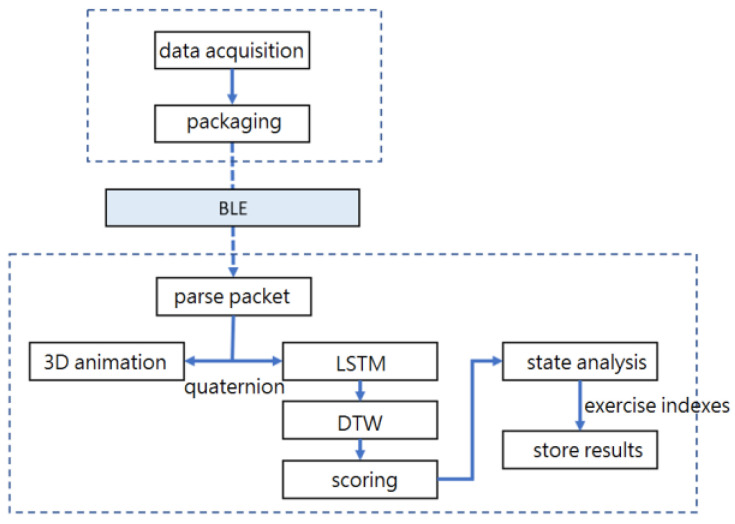
Software architecture.

The software of this system dealt with data acquisition, data packaging, data transmission to the smartphone, motion recognition processing, and user interface display. Figure 6 shows the software architecture.

**Figure 7:**
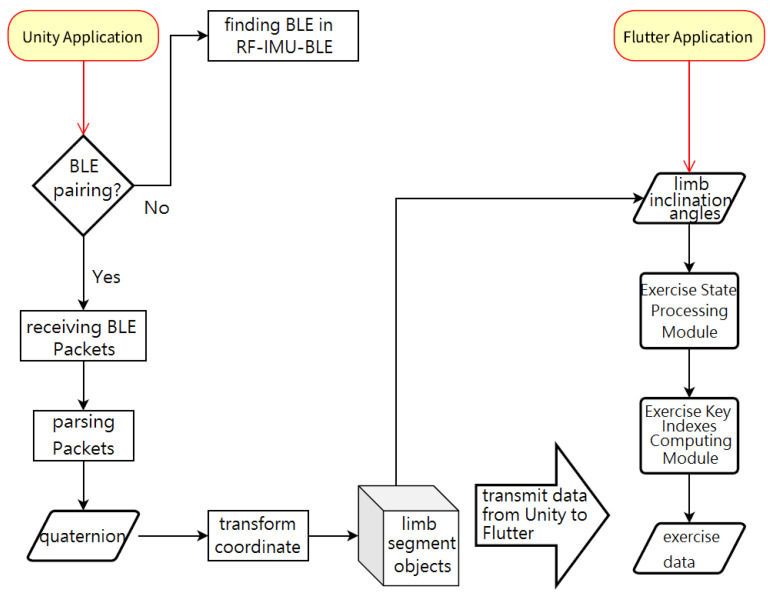
Unity Application and Flutter Application.

The smartphone App was designed using Flutter (ver. 3.3.0), where the Dart programming language was used, and a 3D humanoid avatar was developed through Unity’s Animation System. The Unity Application handles BLE communication, parsing data, motion capture algorithms, and virtual 3D humanoid avatars. The Flutter Application processes activity recognition, motion status detection, exercise key indexes, and database storage. Figure 7 illustrates the relationship between these two Applications.

**Figure 8:**
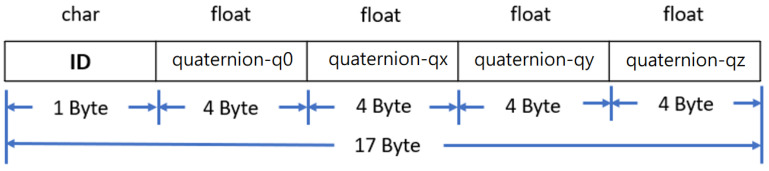
Motion data format.

**Figure 9:**
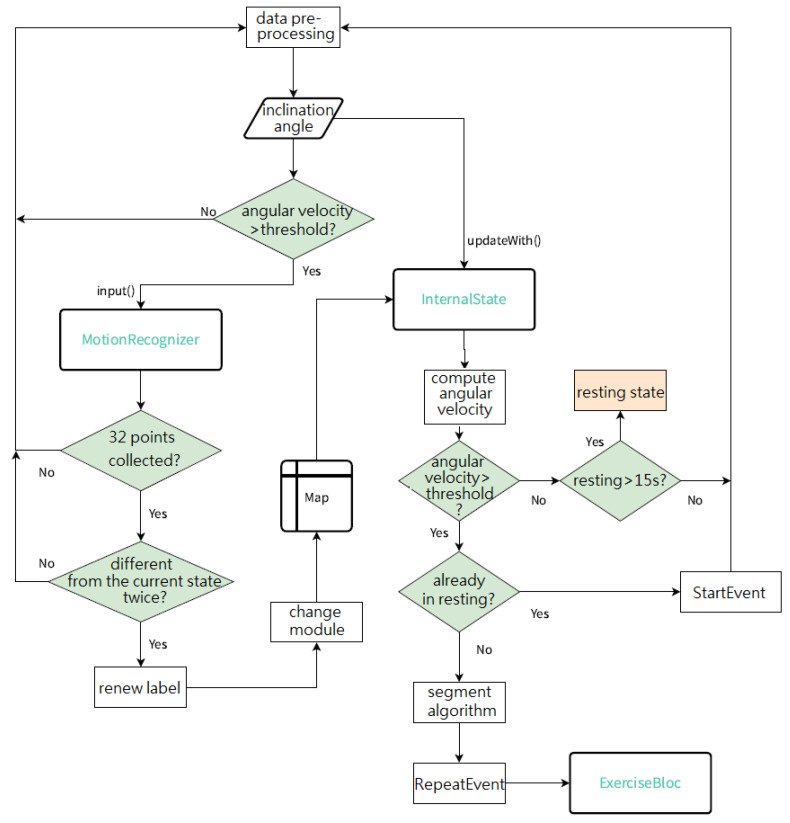
The flowchart of motion data processing.

The purpose of data acquisition is to obtain motion data from RF-IMUs and RF-IMU-BLE. The MCU in the RF-IMU reads the IMU motion data and transmits the data to the RF-IMU-BLE via the RF module. The motion data format is shown in Figure 8. The RF module in the RF-IMU transmits data, while the RF module in the RF-IMU-BLE receives data, forming unidirectional communication. The MCU on the BLE packages and transmits the IMU data from five motion sensing devices (4 RF-IMUs and 1 RF-IMU-BLE) to the smartphone via BLE. The motion data processing is divided into motion event processing and activity recognition, the flowchart of which is shown in Figure 9.

Once the App starts, the limb segment inclination angle is first calculated based on the quaternion from the corresponding IMU. The inclination angle is the angle between the limb segment vector and the vector perpendicular to the horizontal plane. Then, an independent motion event handling module (InternalState) for each motion item is generated, and the angular velocity of the joint is estimated by dividing the inclination angle difference between two consecutive time points by the time interval. The condition of whether the angular velocity exceeds a threshold value is used to determine if the state of the user is in a static or exercise state.

#### 2.2.1. MotionRecognizer

If the user is in an exercise state, the fitness activity recognition (FAR) algorithm (two-layer LSTM model) is activated to determine the current user’s activity type; otherwise, FAR is not executed.

Two-layer LSTM is based on a supervised deep neural network model to train a model for classifying 3 fitness activities. Each sample point contains 4 features (left/right lower-limb angles and left/right upper-limb angles), and the sampling rate is 60 Hz.

**Figure 10:**
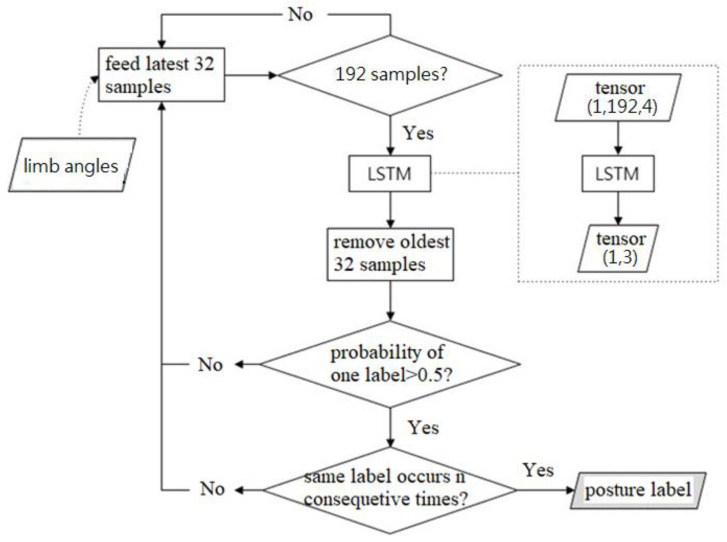
Flowchart of MotionRecognizer.

The training of a two-layer LSTM model was performed on a PC, and then TensorFlow Lite was utilized to convert the trained model to a TensorFlow Lite model file (.tflite) with a smaller size. The TensorFlow Lite model was then deployed on the smartphone, and the IMU data were fed as input data to the lite-trained model on the smartphone for classifying the user’s fitness activities as follows: squats, high knees, and lunges. The flowchart of MotionRecognizer is shown in Figure 10.

#### 2.2.2. InternalState

A new instance object of the InternalState object is also created whenever a new motion starts. Each fitness activity has its own motion event handling, which is stored in the Map object. As the name suggests, the Map is like a map. The Map is a String type parameter as a key, and there are N InternalState modules stored in the Map. Each key is mapped to a separate InternalState object, and the corresponding InternalState object can only be found by using the right key (motion item title).

Based on the current fitness activity type identified by MotionRecognizer, the corresponding exercise state processing is called out from the Map to InternalState and then to ExerciseBloc to calculate key exercise indexes, including the number of sets, reps, training volume, calories burned, power, maximum explosive force, etc.

#### 2.2.3. Segment Algorithm

**Figure 11:**
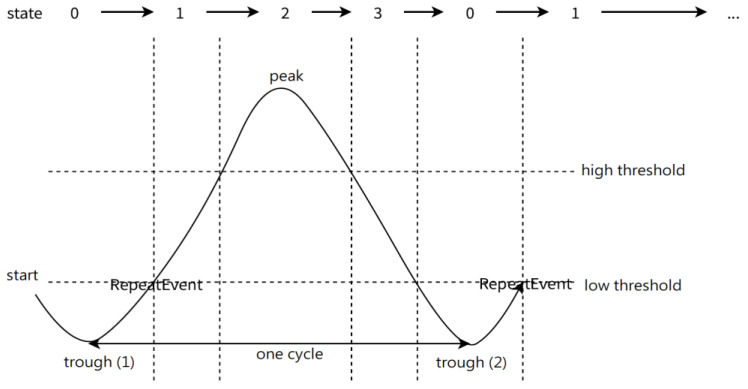
Illustration of segment states.

The inclination angles of the lower and upper limbs of the two legs are fed into the detection function, and the angle of the lower or upper limbs is used as the feature according to different activities. Figure 11 shows the illustration of segmentation.

In state 0, the inclination angle is lower than the low threshold, and which foot meets this condition first is determined. If it is the left foot, the repetition is considered to start with the left foot, and the angle of inclination of the left foot is used as the basis of judgment in the following states (exception: a squat is a synchronized exercise, the angles of inclination of both feet are used as the basis of judgment). Then, after finding the minimum value of the trough (1) in state 0, RepeatEvent will be triggered when the inclination angle exceeds the low threshold value. The work, the power, and the time period between the last trough and the current trough are calculated, and the state becomes state 1.

State 1 becomes state 2 when the inclination angle exceeds the high threshold value, and then state 2 becomes state 3 when the inclination angle is lower than the high threshold value. The maximum peak during state 2 can be found. State 3 returns to state 0 when the inclination angle is lower than the low threshold, and after finding the minimum value of trough (2), RepeatEvent is triggered again when the inclination angle exceeds the low threshold value. The whole process repeats itself.

#### 2.2.4. ExerciseBloc

The ExerciseBloc module calculates the key exercise indexes. These indexes include the number of sets, reps, training volume, calories burned, power, maximum explosive force, and other exercise data.

#### 2.2.5. Dynamic Time Warping (DTW) [25]

DTW is a powerful algorithm in the field of signal processing and pattern recognition, particularly suited for comparing and aligning time-series data that may vary in time or speed. Unlike traditional distance measures, DTW accounts for temporal distortions, making it an invaluable tool for analyzing sequences with varying lengths or time scales. Originally developed in the context of speech recognition, DTW has found applications in diverse domains such as bioinformatics, finance, and gesture recognition.

DTW starts by creating a grid or matrix, where each cell represents a pair of points from the two sequences being compared. The distance or cost between each pair of points is computed, reflecting the dissimilarity between them. A cumulative cost matrix is then constructed by summing the local costs along possible alignment paths. This matrix provides a measure of similarity between the two sequences at each point. Dynamic programming is employed to find the optimal alignment path through the cumulative cost matrix. The goal is to minimize the overall cost of aligning the sequences. Once the optimal path is determined, it is traced back through the matrix to identify the aligned pairs of points. The total cost along the optimal path serves as a similarity measure between the two sequences. Lower costs indicate higher similarity.

## 3. Results

### 3.1. Hardware

**Figure 12:**
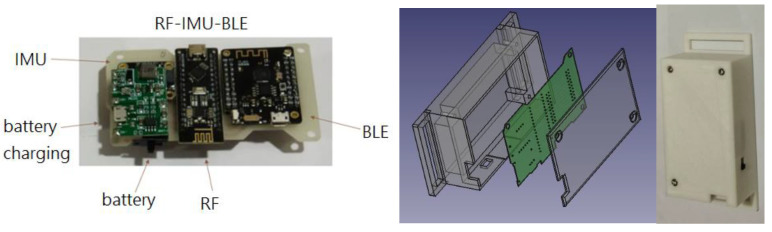
RF-IMU-BLE device.

**Figure 13:**
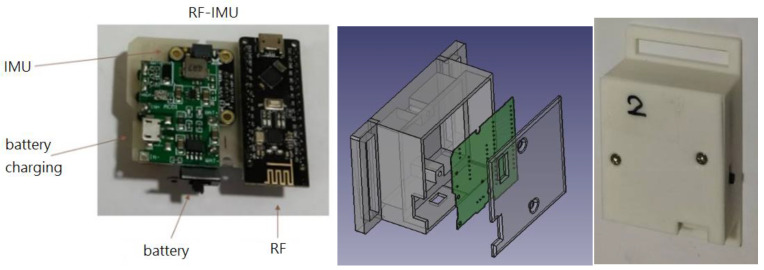
RF-IMU device.

**Figure 14:**
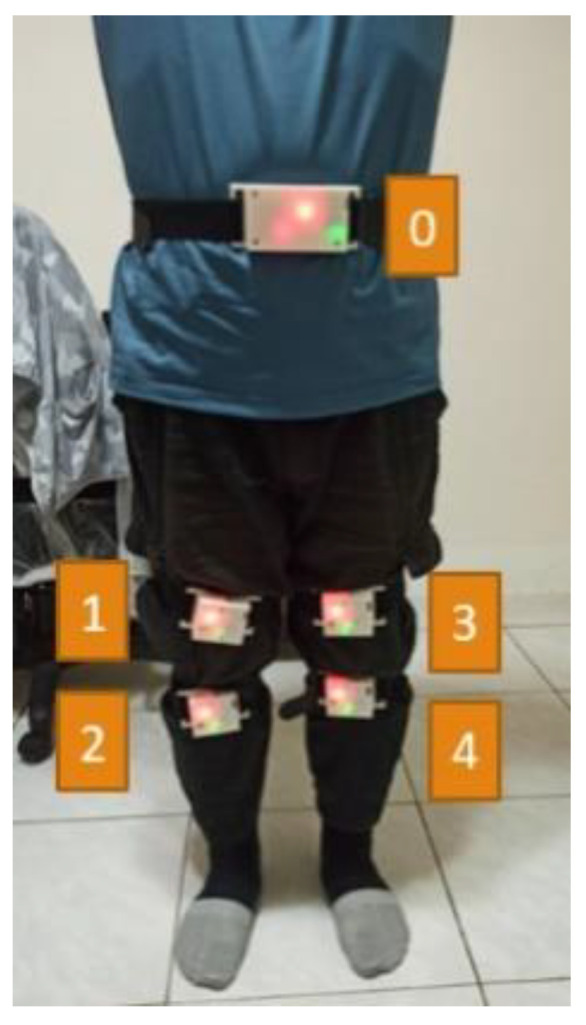
Wearing positions of devices (0: RF-IMU-BLE, 1–4: RF-IMU).

The internal dimensions of the cases for the wearable devices were determined by the sizes of integrated modules. FreeCAD drawing software (ver. 0.21.1) was used to design these cases, and the prototypes were produced using a 3D printer. The RF-IMU-BLE device (ID 0) integrates a microcontroller module with RF communication, a microcontroller module with BLE, an IMU, a lithium battery charging module, and a lithium battery, as shown in Figure 12. The RF-IMU device integrates a microcontroller module with RF communication, an IMU, a lithium battery charging module, and a lithium battery, as illustrated in Figure 13. There are four RF-IMUs (ID 1–4). These five devices together form a wireless wearable lower limb motion capture system. Figure 14 depicts the wearing positions.

**Figure 15:**
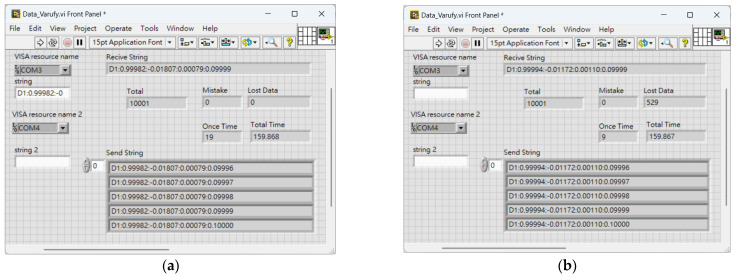
RF transmission performance test result of device ID 1 under two scenarios. (**a**) Scenario 1; (**b**) Scenario 2.

We designed a data transmission test program using LabView (ver. 18.0) to test the wireless transmission performance of the designed wearable devices. The performance refers to the data transmission rate (including the rate of Bluetooth data transmission from the wearable device to the smartphone, and the RF transmission rate between devices), data loss rate, and error rate. The test interface is shown in Figure 15. Two different transmission scenarios were tested. In the first scenario, only one RF device sends data, and in the second scenario, all four RFs send data but the RF in the RF-IMU-BLE device only receives data from one specific RF. The test for the second scenario aims to determine the communication quality under interference. Therefore, the test program only focuses on the performance of receiving one of the four RF devices (ID 1–4) by the RF device (ID 0) at a time. After the experimental tests are completed, the results are averaged to determine the data transmission performance of the wearable device.

There are four RF devices on the lower limbs, which only transmit data and do not need to receive data, so their listening function is turned off to form unidirectional data transmission and the Ack mechanism is canceled. Furthermore, the RF on the abdomen is in pure receiving mode, and the RF in the lower limb devices is changed to pure transmitting mode so that the transmission rate can be increased. In addition, the I2C transmission speed is also reset from 100 Kbps to 800 Kbps to shorten the transmission time of IMU. In order to enhance the efficiency of transmitting the received data from the five IMUs to the Bluetooth processor in the abdominal sensor, we interspersed the quaternion of the five IMUs in the idle time after the reception of RF data and sent it to Bluetooth five times.

Due to the space limitation of this paper, only the performance of the RF of ID 1 is shown here in Figure 15. The performance tests for the RFs of ID 2–4 were all conducted. The RF transmission rate can reach an average of one data transmission every 16 ms. In the second scenario, the average data loss rate is 5.27% and the error rate is 0. The upload data rate of Bluetooth is about 30 ms for five single IMU quaternion transmissions. Since the Bluetooth chip is operated by an independent MCU, neither scenario 1 nor scenario 2 affects its rate. Although the Ack mechanism of RF is canceled, after 80,000 data transmissions, no error data were found. Therefore, even if Ack is canceled, the error rate is still very low.

The verification test proposed in our previous work [16] demonstrated that the designed IMU devices have a mean absolute error of 1.195 degrees for the Z-axis rotation angle and a mean absolute error of 1.485 degrees for the quaternions, proving the quaternion values obtained from the nine-axis sensor (BNO055) are within an acceptable engineering criterion.

### 3.2. Real-Time Motion Display Interface

**Figure 16:**
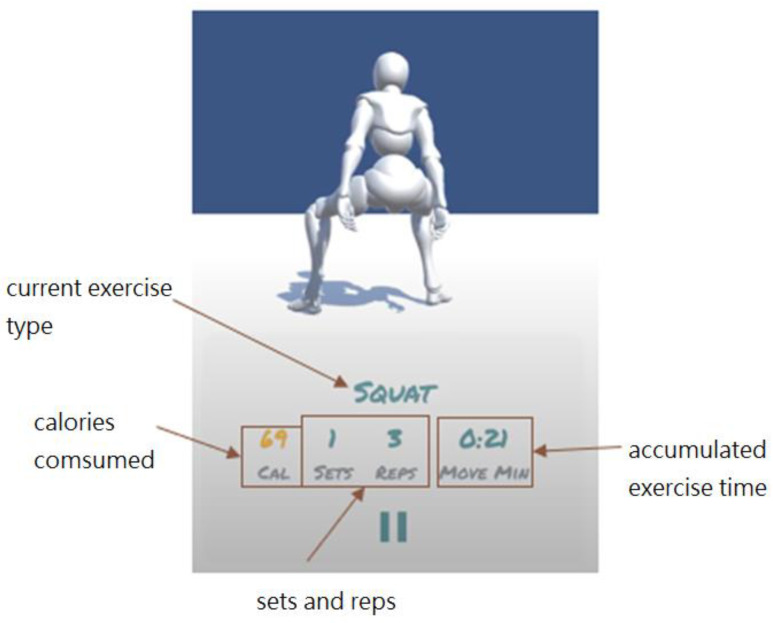
Humanoid avatar screen.

**Figure 17:**
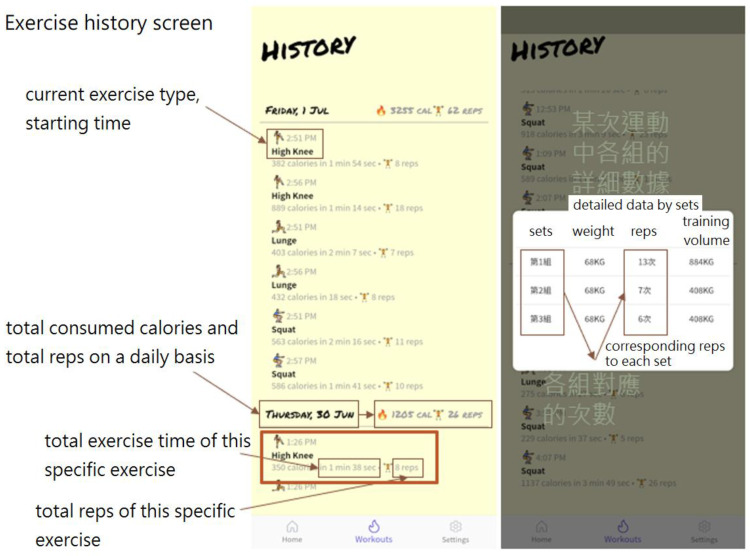
Exercise history screen.

The user interface of the smartphone App consists of the following two main pages in addition to basic login and communication set-up functions:

1. A humanoid avatar screen, as shown in Figure 16: This screen automatically displays the real-time humanoid avatar movements and fitness activity data, including the current activity recognition result, number of sets, number of repetitions, calories consumed, and accumulative exercise time. The activity recognition is based on the TensorFlow Lite model deployed on the smartphone.
2. An exercise history screen, as shown in Figure 17: This screen allows the user to view the historical exercise records on a daily basis or on a specific exercise.

### 3.3. Fitness Activity Recognition (FAR)-LSTM

A K-fold cross-validation was used to verify the accuracy of the proposed two-layer LSTM model. Five participants (four males and one female, all 21 years old) were involved. Each participant performed high knees, lunges, and squats for five minutes each.

**Figure 18:**
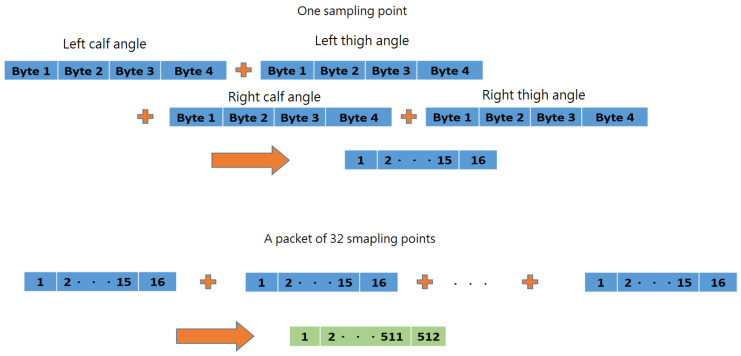
Packaging sampling points.

First, the desired features (left/right thigh/calf angles) were extracted. Each feature uses four single-precision floating point numbers (4 Bytes). There are four features per sampling point and, therefore, 16 Bytes per sampling point. Thirty-two sampling points were packaged into one data set, which is 512 Bytes in total, as shown in Figure 18.

The proposed two-layer LSTM model is a deep neural network, which consists of the first LSTM layer, the first dropout layer, the second LSTM layer, the second dropout layer, and the two hidden layers of 32 and 32 neurons. Since activity recognition is a multivariate classification problem, the number of neurons in the output layer is the number of activity types, which is three in this study. The epoch of the neural network training is 40 (epoch = 40), and the batch size is 64 (batch size = 64).

**Figure 19:**
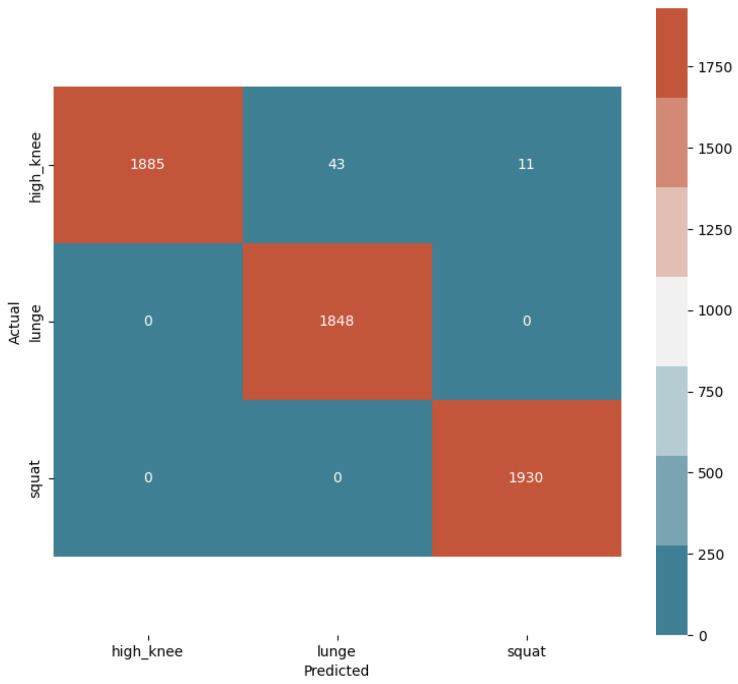
Confusion matrix of the prediction results for participant #3.

Based on K-fold cross-validation, each time, the motion data of four participants are used for training and the motion data of the remaining one participant are used for testing. During training, 190 consecutive sampling points from the training data are packed into one data frame. Each data frame has a 70% overlap. After the model is trained, the sampling points from the testing data are packed to obtain a data frame with a length of 190 sampling points. The data frame is then fed into the trained model to predict the activity type. Figure 19 shows the confusion matrix of prediction results for participant #3. The other confusion matrices are not shown here due to space limitations. Table 2 shows the prediction accuracy of each participant based on K-fold cross-validation. The average accuracy is 97.4%.

### 3.4. DTW

**Figure 20:**
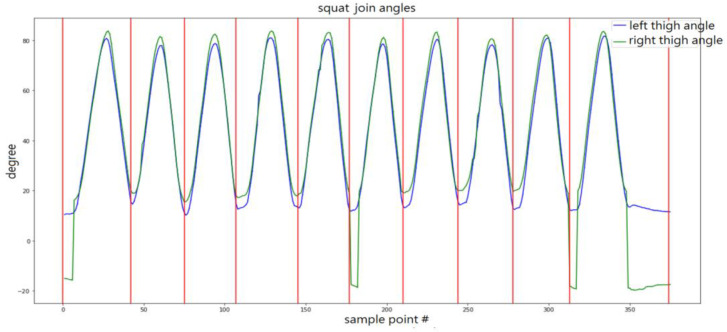
Inclination angles.

**Figure 21:**
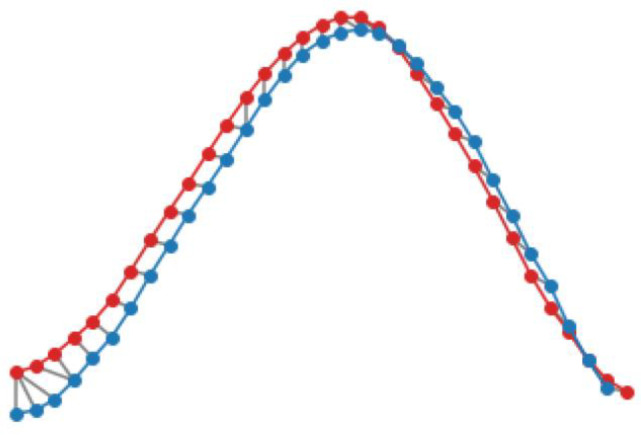
DTW of two cycles for thigh angles (red: coach, blue: trainee).

**Figure 22:**
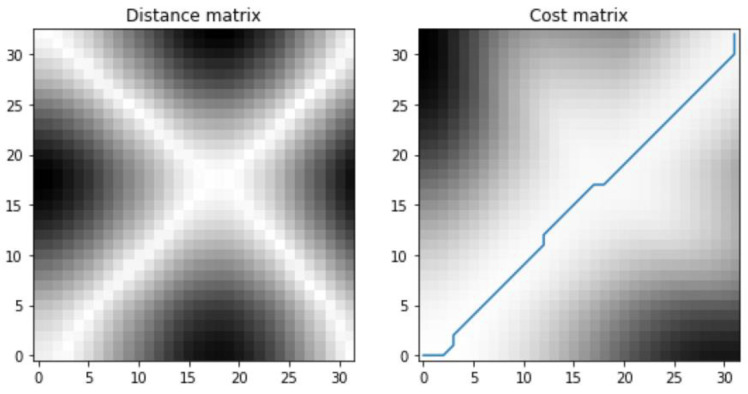
Distance matrix and cost matrix.

The time series of the inclination angles of the left and right thighs were segmented first, and the data from the trough to the trough were defined as a cycle. This is shown in Figure 20. Two different cycles were taken for comparison using DTW alignment, as shown in Figure 21, where the red color represents the data of the coach and the blue color represents the data of the trainee. The distance matrix was calculated first and then the cost matrix was obtained from the distance matrix to find the minimum cost path, as shown in Figure 22. Alignment cost and normalized alignment cost were then calculated. The closer the normalized alignment cost is to 0, the closer the distance between the two waveforms is. For Figure 22, the alignment cost is 50.8305, and the normalized alignment cost is 0.7820 (this value is the similarity score; the lower the better).

## 4. Discussion

The hardware modules used in the proposed system are all available from the shelf and are cost-effective. The motion data measured by IMU were verified within an acceptable engineering criterion. The proposed system can automatically recognize fitness activity types, provide an intuitive virtual humanoid real-time presentation of exercise movements, and calculate various key exercise indicators, offering valuable exercise performance indicators for rehabilitation practitioners or coaches. Particularly, the limb inclination angles calculated in the system are useful for range of motion (RoM) assessments performed by healthcare professionals to evaluate joint and muscle flexibility, help diagnose health conditions, and create effective treatment plans [26].

The problem of RF transmission interference was tackled by adopting unidirectional communication and by designing subtle RF transmission network protocols. The advantage of unidirectional communication is that when an error is detected in the data transmission, the problematic data can be simply ignored. In addition, since the RF module uses the same antenna for both transmission and reception, it needs time to switch between the transmitting and receiving modes. Therefore, instead of spending time handling handshaking protocol to cause data transmission delay, it is better to use unidirectional transmission. The RF transmission rate is up to 16 ms, which may not be suitable for fast sports (e.g., running, etc.), but should be sufficient for slower activities such as rehabilitation or yoga.

The Bluetooth transmission was used to transmit IMU data collected in the RF-IMU-BLE device to the smartphone. The Bluetooth transmission comes with CRC to detect accidental changes in digital data and frequency hopping technology to minimize frequency collision; therefore, the transmission quality of Bluetooth can be trusted.

The information generated by the exercise history screen serves as a valuable reference for tracking an individual’s long-term rehabilitation/fitness progress. These data can be harnessed to create a positive momentum that encourages continued engagement in physical activity. Moreover, the exercise-related insights derived from the designed App can be shared with a social media group, allowing individuals to garner support from friends and relatives.

During the course of this study, we tried one-layer LSTM with the same overlapping percentage. The average accuracy of one-layer LSTM based on K-fold cross-validation is 92.6%, which is lower than that of two-layer LSTM by 4.8%. Since the model training is conducted offline, the extra computational effort would not cost too much. The trained two-layer LSTM model can still be transported to a smartphone using TensorFlow Lite.

The developed system was tested in our laboratory environment in real time. The RF transmission rate of a single RF-IMU can reach an average of 16 ms for transmitting one data set. The sampling rate of 60 Hz is fast enough for capturing slow rehabilitation motion. The upload data rate of the RF-IMU-BLE is about 30 ms for five single IMU quaternion transmissions. The fitness activity recognition (FAR) is based on the TensorFlow Lite model pre-trained on a PC and then deployed on a smartphone. The result of FAR can, therefore, be obtained very fast. Data can be collected to display real-time humanoid avatar movements and fitness activity data.

The recorded movements of the user’s avatar can be replayed, enabling the correction of any incorrect postures during exercise. The DTW provides comparison scores for two similar movements, and this comparison can be made among different lower limb segments. For example, the trainee can compare the angle of his/her left calf to that of the trainer, or the angle of his/her right thigh to that of the trainer.

The proposed system concept can also be applied to arm weight training [16], where IMUs are worn on the upper arm and wrist, but no IMU is worn on the abdomen because the upper torso does not move forward or bend while performing arm weight training. This versatile system also holds potential applications in medical rehabilitation and sports, especially in scenarios where the accuracy of movement is paramount. Our future research will implement and test this system in Taichung Veterans General Hospital and the Fitness Center of the National United University (NUU). The NUU has an In-Body machine [27]. The proposed system, when integrated with In-Body data, encompassing metrics such as weight, muscle mass, body fat percentage, protein levels, bone mass, basal metabolic rate, visceral fat, and total body water content, becomes a comprehensive tool for evaluating fitness performance. Nevertheless, long-term observations are needed to evaluate both at-home rehabilitation and fitness effectiveness.

## 5. Conclusions

This paper focuses on the development of an IMU-based at-home rehabilitation or fitness system, which is low-cost, automatically recognizes the type of exercise, and gathers exercise data into usable metrics. Hardware and software were integrated for this purpose to develop AHRS devices for lower extremity motion capture. A total of five RF-IMU devices were developed. These IMUs were strategically placed, with one on each calf, one on each thigh, and one on the abdomen. A two-layer LSTM model was used for FAR with an average accuracy of 97.4%. An intelligent algorithm was also developed on a smartphone, which performs motion tracking and activity recognition and calculates key exercise variables for three different exercises (squats, high knees, lunges). Upon conducting tests, the developed AI algorithm demonstrates its capability to compute various key exercise indicators in real time. These indicators encompass crucial aspects such as repetitions, intensity, energy consumption, and exercise duration. The algorithm leverages the data generated by users during fitness/rehabilitation to provide instantaneous and personalized insights. Additionally, a 3D humanoid avatar was created on the smartphone App, allowing users to observe their exercise motions in real-time or through replay to track their progress. A DTW algorithm was also integrated into the system for scoring the similarity in two motions. Notably, the proposed RF-IMU-based system stands out for its cost-effectiveness. The adaptability of this system opens up promising applications in both medical rehabilitation and sports, particularly in situations where precision in movement is of importance.

## Figures and Tables

**Table 1:** Unavailable pins in RF-Nano.

GPIO Pin Number (Nano R3)	nRF24L01+ SPI
D9	CE (Chip Enable)
D10	CS/CSN (Chip Select)
D11	MOSI
D12	MISO
D13	SCK

**Table 2:** Accuracy of K-fold cross-validation.

Participant No.	Accuracy
1	0.94
2	0.99
3	0.99
4	0.95
5	1.0

## Data Availability

The datasets presented in this article are not readily available because the data are part of an ongoing study.
